# Comparative Profiling of Ubiquitin Proteasome System Interplay with Influenza A Virus PB2 Polymerase Protein Recapitulating Virus Evolution in Humans

**DOI:** 10.1128/mSphere.00330-17

**Published:** 2017-11-22

**Authors:** Elise Biquand, Juline Poirson, Marwah Karim, Marion Declercq, Nicolas Malausse, Patricia Cassonnet, Cyril Barbezange, Marie-Laure Straub, Louis Jones, Sandie Munier, Nadia Naffakh, Sylvie van der Werf, Yves Jacob, Murielle Masson, Caroline Demeret

**Affiliations:** a Molecular Genetics of RNA Viruses, CNRS UMR 3569, Université Paris Diderot, Sorbonne Paris Cité, Institut Pasteur, Paris, France; b Ecole Supérieure de Biotechnologie Strasbourg, UMR-7242, CNRS, Université de Strasbourg, Illkirch, France; University Medical Center Freiburg

**Keywords:** comparative interactomics, influenza viruses, ubiquitination, virus-host interactions

## Abstract

The optimized exploitation of cell resources is one cornerstone of a successful infection. Differential mapping of host-pathogen protein-protein interactions (PPIs) on the basis of comparative interactomics of multiple strains is an effective strategy to highlight correlations between host proteome hijacking and biological or pathogenic traits. Here, we developed an interactomic pipeline to deliver high-confidence comparative maps of PPIs between a given pathogen and the human ubiquitin proteasome system (UPS). This subarray of the human proteome represents a range of essential cellular functions and promiscuous targets for many viruses. The screening pipeline was applied to the influenza A virus (IAV) PB2 polymerase proteins of five strains representing different levels of virulence in humans. An extensive PB2-UPS interplay has been detected that recapitulates the evolution of IAVs in humans. Functional validation with several IAV strains, including the seasonal H1N1_pdm09_ and H3N2 viruses, confirmed the biological relevance of most identified UPS factors and revealed strain-independent and strain-specific effects of UPS factor invalidation on IAV infection. This strategy is applicable to proteins from any other virus or pathogen, providing a valuable resource with which to explore the UPS-pathogen interplay and its relationship with pathogenicity.

**IMPORTANCE** Influenza A viruses (IAVs) are responsible for mild-to-severe seasonal respiratory illness of public health concern worldwide, and the risk of avian strain outbreaks in humans is a constant threat. Elucidating the requisites of IAV adaptation to humans is thus of prime importance. In this study, we explored how PB2 replication proteins of IAV strains with different levels of virulence in humans hijack a major protein modification pathway of the human host cell, the ubiquitin proteasome system (UPS). We found that the PB2 protein engages in an extended interplay with the UPS that evolved along with the virus’s adaptation to humans. This suggests that UPS hijacking underlies the efficient infection of humans and can be used as an indicator for evaluation of the potential of avian IAVs to infect humans. Several UPS factors were found to be necessary for infection with circulating IAV strains, pointing to potential targets for therapeutic approaches.

## Abstract (precis)

Influenza A viruses (IAVs) are responsible for mild-to-severe seasonal respiratory illness of public health concern worldwide, and the risk of avian strain outbreaks in humans is a constant threat. Elucidating the requisites of IAV adaptation to humans is thus of prime importance. In this study, we explored how PB2 replication proteins of IAV strains with different levels of virulence in humans hijack a major protein modification pathway of the human host cell, the ubiquitin proteasome system (UPS). We found that the PB2 protein engages in an extended interplay with the UPS that evolved along with the virus’s adaptation to humans. This suggests that UPS hijacking underlies the efficient infection of humans and can be used as an indicator for evaluation of the potential of avian IAVs to infect humans. Several UPS factors were found to be necessary for infection with circulating IAV strains, pointing to potential targets for therapeutic approaches.

## INTRODUCTION

The typical virus life cycle sequentially involves cell entry, viral genome uncoating, transcription, replication, protein expression, particle assembly, and egress, along with immune evasion. These processes require a large subset of the host cell machinery. Accordingly, viruses’ capacity to infect, replicate in, and propagate in a given host depends on their ability to appropriately subvert cell pathways and processes. Interaction proteomics, so-called interactomics, is a straightforward approach used to assess virus hijacking of the cellular proteome and decipher its impact on cellular functions. In addition, the differential mapping of host-pathogen protein-protein interactions (PPIs) based on comparative interactomics of multiple strains is an effective strategy to highlight correlations between host proteome hijacking and pathogenesis. The human ORFeome v8.1 (Center for Cancer Systems Biology [CCSB]), a cDNA collection assembling over 11,149 of the estimated 20,000 protein-coding genes existing in the human genome, enables mapping of virus-host PPIs targeted on subarrays of the human proteome (1–3).

In this study, we focused on the ubiquitin proteasome system (UPS), a major pathway of protein posttranslational modifications. Protein ubiquitination involves the stepwise action of three types of enzymes, the E1 ubiquitin-activating enzymes, the E2 ubiquitin conjugating enzymes, and the E3 ubiquitin ligases (4). The E3 ligases perform the ultimate transfer of ubiquitin molecules to substrate proteins and represent the most extended UPS families, consisting of about 1,000 factors (5). Two distinct classes of E3 ligases can be distinguished, i.e., the non-cullin-based E3 ligases that carry out substrate recognition and ubiquitin ligase activity in a single protein and the cullin-based RING domain E3 ligases (CRL) that act as protein complexes. In addition, protein ubiquitination can be reversed by deubiquitinases (DUBs). Ubiquitination determines the stability, activity, or subcellular localization of targeted proteins (6, 7). It is a highly versatile posttranslational modification and is tightly regulated. Hijacking of the UPS seems inherent to the replication cycles of viruses and other intracellular pathogens that manipulate protein ubiquitination to their advantage in various ways to overcome host cell defense mechanisms, cell cycle control, or cell death (8–12). The binding of host UPS factors may also induce the degradation or inhibit the function of viral proteins, thereby taking part in cellular antiviral response processes that constrain viral infection (13–15).

We describe here a systematic comparative interactomics strategy developed to explore the interplay of multiple pathogen proteins with the human UPS and its application to the influenza A virus (IAV) PB2 replication protein. The involvement of UPS in influenza virus infection is supported by an increasing number of studies focused primarily on viral entry/uncoating and escape from cellular antiviral responses (16–19). A functional interplay between the human UPS and viral replication proteins PB1, PB2, PA, and NP (nucleoprotein) has also been reported, and several underlying PPIs have been detected (13, 20–23). In addition, these replication factors have been shown to be ubiquitinated during IAV infection, resulting in increased viral polymerase activity (24). The mechanisms of ubiquitination have been elucidated very recently for NP (23) but remain uncharacterized for PB1, PB2, and PA, constituting the trimeric RNA-dependent RNA IAV polymerase.

The PB2 protein is a key player in IAV pathogenicity (25). From the crystal structure of the viral polymerase complex, PB2 appears to provide large, flexible, and shape-changing interfaces accessible for interactions with host proteins (26, 27). The comparative UPS-PB2 interactomic analysis was performed with the PB2 proteins of five IAV strains with various degrees of virulence to evaluate the correlation with IAV pathogenic power in humans. A split-luciferase complementation assay (high-throughput *Gaussia princeps* protein complementation assay [HT-GPCA]) (28) was used as a systematic PPI screening method against a targeted library covering about half of the whole human UPS. A high-confidence set of PB2-UPS interactions has been identified. The interaction profile-based clustering segregated according to the level of IAV strain adaptation to humans. Functional validation with three IAV strains indicated the biological relevance of identified UPS factors to IAV infection in a common or strain-specific manner. This strategy, applicable to any other pathogen proteins, will certainly prove effective for pinpointing relationships of UPS hijacking with pathogenic power in other microbial systems.

## RESULTS

### The UPS-targeted library.

**FIG 1:**
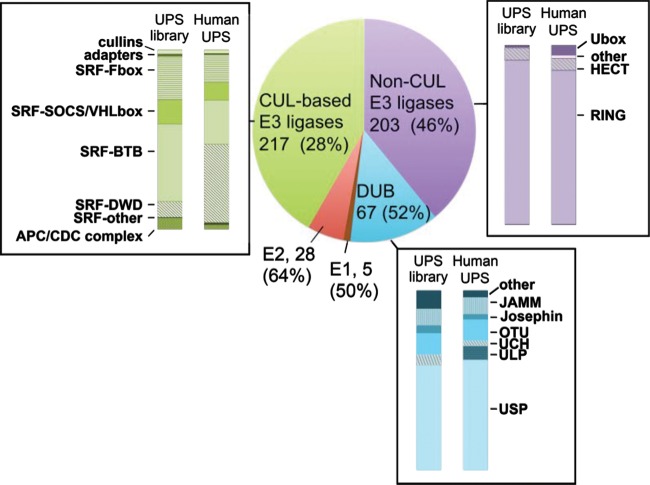
Pie chart representation of the UPS library. For each category of the UPS, the number of factors is indicated, as is the coverage of the *in silico* human UPS database (in parentheses). The representativeness of subcategories for the DUB, cullin-based (CUL-based), and non-cullin-based (Non-CUL) E3 ligases is shown for the human UPS and the UPS library.

A UPS library suitable for PPI detection via the HT-GPCA method was first constructed by assembling 590 cDNAs encoding 558 unique human UPS factors (see Table S1 in the supplemental material) cloned in fusion with the N-terminal Glc1 fragment of the *G. princeps* luciferase (29). The coverage of this library was estimated in comparison with an *in silico* human UPS database, designated the Human UPS, that was obtained by combining the existing DuDe and UUCE ubiquitin-dedicated databases (5, 30) (Table S1). Our UPS library covers 50% of the human E1-activating enzymes, 64% of the E2 ubiquitin-conjugating enzymes, 46% of the non-cullin-based E3 ubiquitin ligases, 28% of the cullin-based E3 ubiquitin ligases, and 52% of the DUBs (Fig. 1). With few exceptions (E3-DWD, DUB-ULP), the distribution of the different subcategories of E3 ubiquitin ligases and of DUBs in the UPS library is comparable to that in the Human UPS (Fig. 1). This is an important feature, since the E3 and DUBs are the major regulators of protein ubiquitination/deubiquitination and thus are prime targets of viruses.

10.1128/mSphere.00330-17.5 TABLE S1  The UPS library compared to the *in silico* human UPS database, corresponding to the combined UUCD-DuDe databases. Gene symbols are shown. The UPS categories are derived from the UUCD 2.0 database. Download TABLE S1, XLSX file, 0.1 MB. Copyright © 2017 Biquand et al. 2017 Biquand et al. This content is distributed under the terms of the Creative Commons Attribution 4.0 International license.

### Implementation of HT-GPCA for detecting UPS-PB2 PPIs.

The HT-GPCA method relies on the ability of interacting protein pairs expressed in fusion with the Glc1 and Glc2 complementary fragments of the *G. princeps* luciferase to reconstitute an active enzyme. We first evaluated whether this assay could successfully distinguish between interacting and noninteracting pairs when applied to the PB2 proteins from IAV. Since the UPS factors are fused to the Glc1 fragment of the luciferase, the PB2 proteins had to be fused to the complementary Glc2 fragment. The PB2 proteins used for HT-GPCA evaluation were the ones chosen for the comparative screening with the human UPS, originating from five IAV strains with various degrees of virulence in humans, i.e., laboratory-adapted strain H1N1_WSN_ (A/WSN/33), low- to mild-virulence human seasonal viruses H1N1_pmd09_ (A/Bretagne/7608/2009) and H3N2 (A/Center/1003/2012), and highly virulent viruses H1N1_1918_ (A/Brevig Mission/1/1918) and H7N9 (A/Anhui/1/2013).

Interactions between the PB2 proteins fused to Glc2 at the N- or C-terminal position were assessed with a positive reference set (PRS) of proteins known from the literature to interact with PB2 (31) and a random reference set (RRS) of proteins *a priori* not interacting with PB2. The PRS and RRS gave distinct ranges of signals in both Glc2 fusion configurations (Fig. S1). Some overlap exists between the higher RRS and lower PRS signals, which was expected because some RRS proteins were known to have strong proaggregation properties (31). In addition, the interactions of several of the PRS proteins chosen (NPM1, NFX1, RAB11A) with PB2 turned out to be poorly detectable by HT-GPCA (Table S2) but still stronger than those of most of the RRS proteins.

10.1128/mSphere.00330-17.1 FIG S1  Comparison of the two fragment positions in the fusion with the PB2 proteins for HT-GPCA. (a) HEK-293T cells were transfected with a set of plasmids expressing, fused to the Glc1 fragment of the *G. princeps* luciferase, proteins known to interact with PB2 (PRS) or randomly chosen as *a priori* not interacting with PB2 (RRS) and PB2 proteins fused to the Glc2 fragment at the C or N terminus. Luciferase signals were measured at 24 h posttransfection. *P* values were calculated with a Wilcoxon test. *, *P* <0.05; **, *P* <0.01; ***, *P* <0.001; ****, *P* <0.0001. (b) Schematic of the Glc fusions used for screening. The domains of the full-length *G. princeps* luciferase ORF are represented at the top. (c) Expression levels of the Glc2-PB2 proteins in HT-GPCA settings. The Glc2-fused PB2 proteins in whole-cell lysates of HT-GPCA samples were analyzed by Western blotting with rabbit anti-*Gaussia* antibodies, and an anti-tubulin antibody was used as a loading control. Download FIG S1, EPS file, 0.6 MB. Copyright © 2017 Biquand et al. 2017 Biquand et al. This content is distributed under the terms of the Creative Commons Attribution 4.0 International license.

10.1128/mSphere.00330-17.6 TABLE S2  Comparison of the Gluc2 fragment position in fusion with the PB2 proteins for HT-GPCA. Download TABLE S2, XLSX file, 0.05 MB. Copyright © 2017 Biquand et al. 2017 Biquand et al. This content is distributed under the terms of the Creative Commons Attribution 4.0 International license.

The PB2-Glc2 C-terminal fusions generated stronger signals with the nuclear pore (NUP50, NUP62) and nuclear import (KPNA1) factors, associating with the extreme C-terminal nuclear localization signal motif of PB2. Both Glc2 configurations worked similarly with the other PRS proteins (Table S2). We chose to use the Glc2-PB2 N-terminal fusions for PPI screening, reasoning that adding a Glc2 tag at the N terminus of PB2 would better comply with the PB2 C-terminal domain’s flexibility that appeared from the crystal structure of the polymerase (27).

The overall levels of luminescence varied according to the PB2 proteins but were not related to differences in Glc2-PB2 protein accumulation (Fig. S1). They likely reflect differences in the intrinsic binding properties of the five PB2 proteins.

### HT-GPCA screening of PB2-UPS interactions.

**FIG 2:**
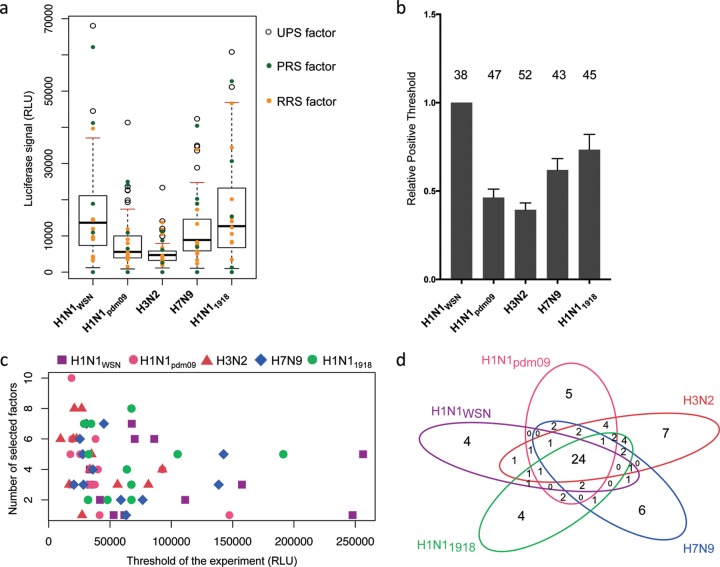
First screening of the UPS library with PB2 proteins. (a) Example of UPS factor screening in the first HT-GPCA experiment. For each PB2 protein, whisker plots were generated from the luminescence values of the UPS-PB2 pairs. The PT for each PB2 is red. Outlier luminescence values, represented by open circles, were selected as potential positive interactions. The RRS and PRS values (yellow and green dots, respectively) were plotted over the whisker plots afterward to evaluate the accuracy of the outlier-based selection. (b) PTs obtained in each individual HT-GPCA experiment were calculated relative to the PT of H1N1_WSN_ PB2. The total number of UPS factors selected in the first screening is shown at the top of each histogram for each PB2 protein. (c) Number of selected factors according to the PT in each experiment for the five PB2 proteins. There is no correlation between the PT and the number of factors selected (Pearson’s product-moment correlation test: *R*^2^ = −0.2207, *P* value = 0.09015). (d) Venn diagram indicating the numbers of common UPS factors identified with the different PB2 proteins. RLU, relative luminescence units.

The entire UPS library screening was conducted via 12 independent HT-GPCA experiments wherein a set of 25 to 72 exploratory Glc1-UPS factors were screened for interactions with the five individual Glc2-PB2 proteins along with the PRS and RRS proteins (Table S3). In each experiment, a positive threshold (PT) was calculated for each PB2 protein on the basis of the distribution of the luminescence values generated by the PB2-UPS pairs. The PT corresponded to the third quartile plus 1.5 times the interquartile space [PT = Q3 + (1.5 × IQR)]. UPS factors generating outlier luminescence values above the PT were selected as potential interacting partners (Fig. 2a; Fig. S2). This selection was validated by the distribution of the PRS and RRS proteins relative to the PT (Fig. 2a; Fig. S2). A fraction of the PRS proteins consistently generated luminescence values under the PT (Table S3), indicating the high stringency of the selection of positive interactions from this initial screening with the defined PT.

10.1128/mSphere.00330-17.2 FIG S2  UPS library screening. The 12 HT-GPCA experiments covering the screening of the whole UPS library are shown. In each experiment, whisker plots were generated from the luminescence values of the UPS-PB2 pairs. Outlier luminescence values, represented by circles, were selected as potential positive interactions. The RRS and PRS values (yellow and green dots, respectively) were plotted in the whiskers plots afterward to evaluate the accuracy of the outlier-based selection. Download FIG S2, EPS file, 0.9 MB. Copyright © 2017 Biquand et al. 2017 Biquand et al. This content is distributed under the terms of the Creative Commons Attribution 4.0 International license.

10.1128/mSphere.00330-17.7 TABLE S3  Raw *Gaussia* activities obtained in the 12 HT-GPCA experiments performed for the first PB2-UPS PPI screening. Download TABLE S3, XLSX file, 0.1 MB. Copyright © 2017 Biquand et al. 2017 Biquand et al. This content is distributed under the terms of the Creative Commons Attribution 4.0 International license.

While the PTs differed among the various PB2 proteins, their relative levels were preserved between experiments (Fig. 2b), according to differences in PB2 intrinsic binding properties detected with the PRS and RRS proteins (Fig. S1). This disparity did not alter the sensitivity with which GPCA identified potential interacting partners, since the number of UPS factors selected was independent of the PT level of the PB2 proteins (Fig. 2c). The initial PB2-UPS screening, where each UPS factor was screened once with the PB2 proteins, identified 91 UPS clones, corresponding to 80 unique UPS proteins, as putative interacting partners of at least one PB2 protein (Table S2). Twenty-four factors were selected with all five of the PB2 proteins, whereas the remaining 56 factors exhibited distinct interaction patterns between strains (Fig. 2d).

### Postscreening retesting of the PB2-UPS interacting pairs.

The interactions selected from the initial screening were retested by applying the NLR (normalized luminescence ratio) method to the HT-GPCA (28). This method, taking into account the background interaction level of the Glc1 or Glc2 fusion partner, has been shown to accurately discriminate PB2 interacting partners from noninteracting proteins in the context of infection (31). To improve assay detectability and to allow a parallel comparison, each of the 80 UPS factors selected was retested against all five PB2 proteins in three biological replicates (Table S4). For each PB2 protein individually, the NLRs obtained with the set of 11 RRS proteins were used to calculate a 99.73% confidence interval. The upper limit of this confidence interval was used as the threshold for the selection of positive interactions (Table S4). By this selection strategy, 42 UPS factors scored >3 three times with at least one PB2 and were considered “high-confidence” PB2 interactors.

10.1128/mSphere.00330-17.9 TABLE S4  NLRs obtained in the retesting experiments with selected UPS factors. The NLR threshold for positive interactions is shown at the bottom of each column. The high-confidence UPS factors considered validated are shown on the right. Download TABLE S4, XLSX file, 0.1 MB. Copyright © 2017 Biquand et al. 2017 Biquand et al. This content is distributed under the terms of the Creative Commons Attribution 4.0 International license.

### Hierarchical clustering of PB2-UPS interaction profiles.

**FIG 3:**
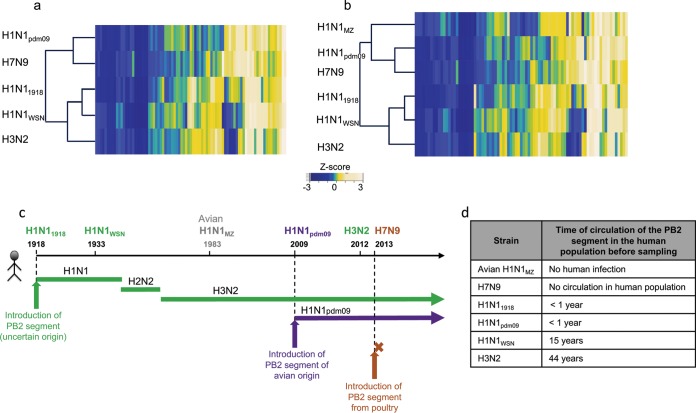
Interaction dendrograms deduced from PB2-UPS interaction mapping. (a) Hierarchical clustering of the five PB2 proteins indicated on the basis of their human UPS interaction profiles. Heat maps represent the *z* scores derived from an experimentally homogeneous set of NLRs obtained by the retesting of 75 UPS factors at once in the NLR retesting-2 experiment (Table S3). (b) Hierarchical clustering of PB2-UPS interaction profiles including H1N1_MZ_ PB2. (c) Schematics delineating, for the IAV strains used in this study the year of PB2 segment introduction into humans (vertical arrows). The brown arrow indicates the infection of a human patient without any spread in the human population; the course of the PB2 segment in the human population is indicated by plain lines (adapted from reference 41). (d) Time of circulation in the human population of the PB2 segment before strain sampling.

PB2-UPS interaction profiles were determined by using *z* score transformation of the NLR. They were compared by agglomerative hierarchical clustering among the different strains (see Materials and Methods), which in turn was used to calculate an interaction dendrogram (Fig. 3a).

PPI profile-based clustering separates viral strains into two groups, i.e., strains recently introduced into the human population from an avian origin (H1N1_pdm09_ and H7N9) and human-adapted strains with a PB2 segment sequentially deriving from the H1N1_1918_ ancestor. The PB2-UPS interaction profiles of the latter group further clustered according to the duration of circulation of the PB2 segment in the human population as follows: <1 year for H1N1_1918_, 15 years for H1N1_WSN_ (followed by adaptation to laboratory cell lines after sampling), and 44 years for H3N2 (Fig. 3c and d). We next analyzed the interaction profile of PB2 from avian strain A/Mallard/Marquenterre/Z2371/83 (H1N1_MZ_) with this set of UPS factors. *z* scores were calculated for the NLR obtained and used to compare the UPS/PB2 H1N1_MZ_ profile to those of the PB2 proteins from five human-infecting strains (Fig. 3b; Table S4). H1N1_MZ_ PB2 grouped with the other avian-origin H1N1_pdm09_ and H7N9 PB2 proteins, but the latter, having recently gained human infection potential, remained closer to each other (Fig. 3b). It can thus be hypothesized from the PB2-UPS interaction dendrogram that the interplay between the PB2 polymerase protein and the human UPS may have evolved along with virus adaptation to humans, i.e., acquisition of the capacity to infect humans from an animal reservoir (H7N9 versus H1N1_MZ_), gaining human-to-human transmission capability (H1N1_pdm09_ versus H7N9) and then duration of circulation in the human population (H1N1_1918_ < H1N1_WSN_ < H3N2).

### Functional impact of PB2 interactors.

To demonstrate the biological relevance of the PB2-UPS PPIs identified, we studied the involvement of the UPS targets of PB2 in the production of infectious viral particles. For that purpose, we examined the productive infectious cycle of seasonal virus strains H1N1_pdm09_ and H3N2 and of the H1N1_WSN_ laboratory-adapted strain upon small interfering RNA (siRNA)-mediated depletion of the individual PB2 interactors. Seasonal strains H1N1_pdm09_ and H3N2 grow poorly in the A549 human pulmonary cell line. We thus used reverse genetics to produce adapted H1N1_pdm09_ and H3N2 viruses harboring an intact PB2 segment and point mutations in the HA segment that efficiently infected A549 cells (see Materials and Methods).

**FIG 4:**
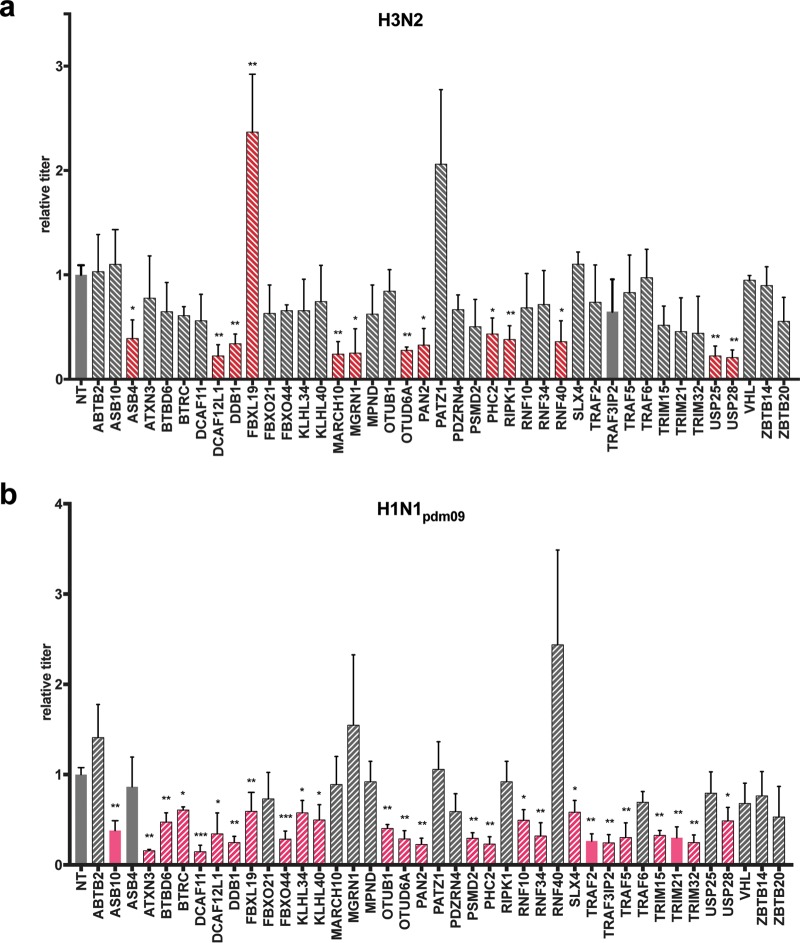
Functional evaluation of the involvement of the UPS PB2 partners in the multiplication of H3N2 and H1N1_pdm09_. A549 cells transfected with nontargeting (NT) or UPS-targeting siRNA for 48 h were infected with H1N1_pdm09_ or H3N2 at an MOI of 0.001 PFU/cell. The titers of the viruses produced were determined in the supernatants collected at 24 h postinfection by plaque-forming assay and expressed as ratios relative to the titers obtained with nontargeting siRNA. UPS factors with statistically significant effects are red (H3N2) or pink (H1N1_pdm09_); hatched bars indicate that a PPI with the corresponding PB2 protein has been detected, taking into account positive scoring at least two times in the NRL retesting experiments; and plain bars indicate that no interaction was detected (validated fewer than two times in NLR retesting). Data represent the mean ± the standard error of the mean of three independent experiments, and *P* values were calculated with a two-tailed nonparametric Student *t* test. *, *P* <0.05; **, *P* <0.01; ***, *P* <0.001.

**FIG 5:**
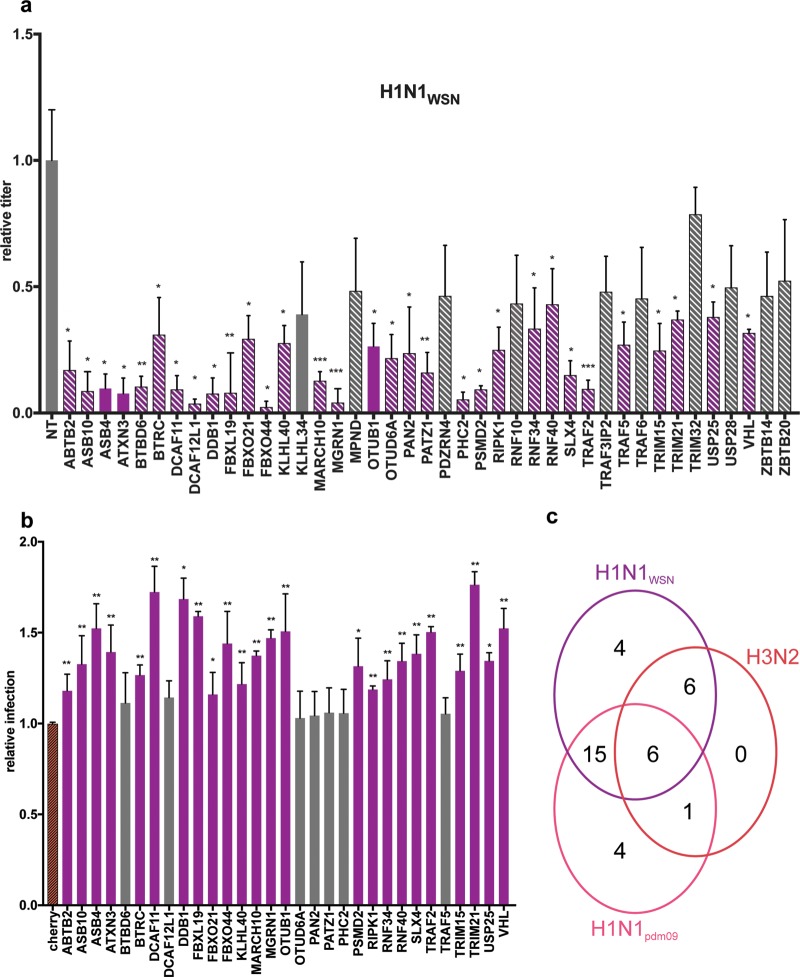
Functional evaluation of the involvement of the UPS PB2 partners in the multiplication of the laboratory-adapted strain H1N1_WSN_ virus. (a) Effect of siRNA-mediated depletion. A549 cells transfected with nontargeting (NT) or UPS-targeting siRNA for 48 h were infected with H1N1_WSN_ at an MOI of 0.0001 PFU/cell for 24 h. Viral titers were determined by plaque-forming assay and are expressed as ratios relative to the titers obtained with nontargeting siRNAs. UPS factors with statistically significant effects are purple, hatched bars indicate that a PPI with H1N1_WSN_ PB2 was detected, and plain bars indicate that no interaction was detected. (b) Effect of UPS factor overexpression. Plasmids expressing the UPS factors fused to mCherry were transfected into HEK-293T cells. Twenty-four hours after transfection, the cells were infected at an MOI of 1 PFU/cell for 18 h with a reporter H1N1_WSN_ virus expressing the mCitrine fluorescent protein. The percentage of mCitrine-positive infected cells in Cherry-UPS-expressing cells was determined by fluorescence-activated cell sorter analysis and is expressed as a ratio relative to the mCitrine-positive cells expressing unfused mCherry. Data represent the mean ± the standard error of the mean of three independent experiments, and *P* values were calculated with a two-tailed nonparametric Student *t* test. *, *P* <0.05; **, *P* <0.001; ***, *P* <0.001. (c) Venn diagram illustrating the number of UPS factors found to be involved in the multiplication of one or several IAVs.

Forty-one high-confidence PB2 interactors were assessed, while the ubiquitin protein was excluded owing to its pleiotropic function. A549 cells transfected with siRNAs targeting the individual UPS factors or transfected with a nontargeting siRNA were infected for 24 h with the H1N1_WSN_, adapted H1N1_pdm09_, or H3N2 virus. Knockdown efficiency and cell viability were assessed for the different siRNAs (Fig. S3). The titer of infectious IAV particles produced was measured by plaque assay. The effects of UPS factor depletion were calculated by comparing the virus titers produced with those obtained with a nontargeting siRNA. Of the 41 PB2 interactors tested, 36 showed an effect on infection with all or a subset of the IAV strains, albeit to various degrees. Statistically significant (*P* < 0.05) reductions in viral production were observed with the depletion of 13 (H3N2), 26 (H1N1_pdm09_), and 31 (H1N1_WSN_) factors. The decreases ranged between 2.3- and 4.8-fold for H3N2, between 1.7- and 7-fold for H1N1_pdm09_, and between 2.3- and 50-fold for laboratory-adapted strain H1N1_WSN_ (Fig. 4 and 5a; Table S5). The various effects of the siRNAs suggest that the UPS factors identified may have critical roles or modulate IAV infection. Most of the UPS factors that had an effect on viral production were also among those found to bind the PB2 protein of the corresponding strain. The demonstration of the direct role of PB2-UPS interactions will nonetheless require the study of mutations that disrupt PB2-UPS interactions.

10.1128/mSphere.00330-17.3 FIG S3  Toxicity and silencing efficiency of siRNAs. (a) Toxicity of siRNA. A549 cells were transfected with 25 nM siRNA, and cell viability was determined at 72 h posttransfection by using trypan blue. The results are expressed as mean percentages ± the standard error of the mean (*n* = 3). There is no correlation between the slight loss of cell viability observed for several siRNAs and the functional effect of these siRNAs on the virus cycles of the three strains tested. (b) Silencing efficiency of siRNA. A549 cells were transfected with 25 nM control or UPS-targeting siRNA and with plasmids encoding the corresponding UPS protein fused with the full-length *G. princeps* luciferase (pGlcFL-UPS). The ratio of the luciferase activity obtained in cells transfected with the UPS-targeting siRNA to that obtained in cells transfected with the control siRNA is shown. The results are represented as floating bars with a line at the mean. Download FIG S3, EPS file, 0.3 MB. Copyright © 2017 Biquand et al. 2017 Biquand et al. This content is distributed under the terms of the Creative Commons Attribution 4.0 International license.

10.1128/mSphere.00330-17.8 TABLE S5  Relative viral titers produced upon siRNA-mediated depletion of UPS factors. Viral titers were calculated relative to the titer obtained with a nontargeting siRNA. The functional families of the corresponding UPS factors and associated domains are shown. Download TABLE S5, XLSX file, 0.1 MB. Copyright © 2017 Biquand et al. 2017 Biquand et al. This content is distributed under the terms of the Creative Commons Attribution 4.0 International license.

Six UPS factors were discovered to be essential for infection with the three viruses (DCAF12L, DDB1, FBXL19, OTUD6A, PAN2, and PHC2) (Fig. 5c; Table S5). These functional studies also identified four factors (TRIM32, TRAF3-IP2, RNF10, and KHLH34) that specifically modulate infection with the H1N1_pdm09_ virus from the 2009 pandemic.

The UPS factors that were found to mediate H1N1_WSN_ infection were ectopically expressed fused to the mCherry protein to evaluate the effect of their overexpression on infection with recombinant H1N1_WSN_ virus expressing the mCitrine fluorescent reporter (Fig. 5b). Twenty-four factors increased viral replication, as measured by the percentage of mCitrine-positive cells (Fig. 5b), further validating their role in IAV infection.

The functional evaluation revealed that UPS factors may have strain-independent or strain-specific effects on IAV infection, highlighting a differential involvement of the UPS in IAV infection (Fig. 5c).

## DISCUSSION

The split-luciferase strategy developed here for the comparative mapping of interactions between the IAV PB2 protein and factors of the human UPS shows a number of noticeable benefits allowing the straightforward delivery of high-quality, systematic interaction mapping. It takes advantage of the excellent performance of the PPI HT-GPCA for the specific and sensitive detection of PPIs (28, 32, 33). Although the HT-GPCA can tolerate a certain distance between interacting pairs of proteins (28, 34), it is assumed that the use of a single Glc fusion configuration would likely lose some PPIs. For more exhaustiveness, PPI screening should be applied by using two (N and C terminal) or even four (Glc1 and Glc2 both N and C terminal) possible configurations, which is out of scale for high-throughput screenings.

Evaluation of the same matrix of host factors for interactions with multiple pathogen proteins provides rigorous comparative interaction mapping, as applied here to the human UPS and the PB2 protein of IAVs. Importantly, the various intrinsic properties of pathogen proteins are not problematic for the comparative aspect of this interaction mapping, as demonstrated in this study with the PB2 proteins of five different IAV strains.

Paramount issues for this systematic interactomics strategy are the quality and exhaustiveness of the library representing a subarray of the human proteome. The UPS-dedicated library that was assembled and carefully characterized contained 558 unique UPS factors out of the 1,277 described in databases (29), covering one-third to two-thirds of the different UPS categories (E1, E2, various E3 ligase classes). The relative UPS library composition parallels the database-derived human UPS composition, notably regarding the E3 ligases or their substrate recognition factors (SRFs). This is of key importance since E3 ligases give the human UPS its complexity and flexibility in protein targeting and are consequently the major targets of viruses (8). The UPS-dedicated library thus offers an accurate representation of the global human UPS, ready to use for mapping of interactions with any protein by HT-GPCA. The same strategy can be implemented with other subarrays of the proteome, provided that HT-GPCA-compatible libraries are available. Smaller libraries dedicated to the alpha/beta interferon, NF-κB, and transforming growth factor beta pathways actually exist and have been used to benchmark the HT-GPCA by using a human-human protein interaction matrix (28).

The usurpation of UPS is a recognized significant process of viral infections that may underlie essential traits of pathogenic potential (11, 12). An interplay between the IAV replication proteins and the UPS has been detected (13, 20, 22, 23), which is only partially characterized. The comparative interactomics strategy applied in this study addresses this knowledge gap. Indeed, we identified a rather extensive interplay between PB2 and the human UPS and showed that PB2 binds to E3 ubiquitin ligases or E3-related factors (“others” in our UPS library), to DUBs, and to a proteasome subunit but not to E1 or E2 enzymes. Moreover, the PB2 proteins interact with all categories of E3 ligases, RING domain ligases as well as cullin-based RING domain E3 ligase (CRL) complexes (Table S6). The targeted proteins in those CRL complexes are mainly SRFs, in line with the dysregulation of cellular protein ubiquitination often observed with viruses through UPS hijacking. The targeting of several DUBs also suggests that PB2 might interfere with the versatility of the ubiquitination process. Finally, PB2 appears to broadly target UPS proteins without subcategory preference (Fig. S4), possibly reflecting a wide manipulation of the UPS during IAV infection.

10.1128/mSphere.00330-17.4 FIG S4  Representativeness of the UPS categories. Shown is a pie chart of different UPS categories in the UPS library and in the UPS targets of PB2. Download FIG S4, EPS file, 1 MB. Copyright © 2017 Biquand et al. 2017 Biquand et al. This content is distributed under the terms of the Creative Commons Attribution 4.0 International license.

10.1128/mSphere.00330-17.10 TABLE S6  Protein domains found in the UPS factors validated for their interaction with PB2 and representativeness of the UPS PB2 targets. Download TABLE S6, XLSX file, 0.05 MB. Copyright © 2017 Biquand et al. 2017 Biquand et al. This content is distributed under the terms of the Creative Commons Attribution 4.0 International license.

**FIG 6:**
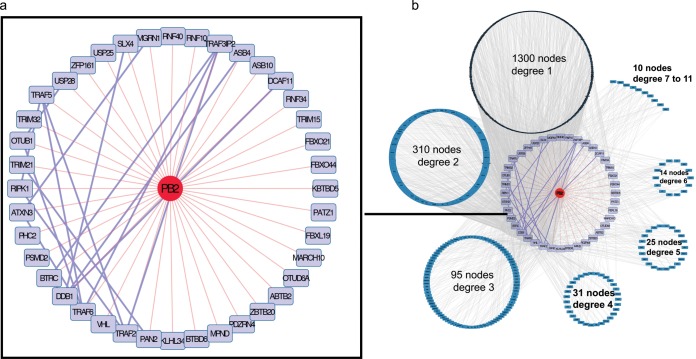
The PB2-UPS interactome. (a) Map of PB2-UPS interactions. Direct PB2-UPS interactions are red and comprise interactions detected with all of the PB2 proteins tested. Interactions between the UPS targets of PB2 are violet. (b) Plugging of the PB2-UPS network into the human interactome. PB2 UPS targets are violet, the host proteins they connect to are organized in circles according to their degree of connection (node degree) with the UPS factors interacting with PB2. A node degree of 1 indicates an interaction with one UPS factor of the PB2-UPS interactome, a node degree of 2 indicates an interaction with two UPS factors of the PB2-UPS interactome, etc.

Virus-host protein interaction networks can be deduced from the binary PPI data sets obtained. A PB2-UPS interaction map encompassing all of the strains tested was plugged into the human interactome recovered from the BioGRID PPI database. Some of the PB2 targets interact with each other (Fig. 6a), underlying an intricate interplay where PB2 binds to UPS proteins that already act in complexes. When all human factors interacting with the UPS partners of PB2 are considered, an elaborate interaction map emerges (Fig. 6b), revealing the possible impact of PB2 on the human proteome through UPS targeting. Affinity purification coupled with mass spectrometry was recently used to determine the comparative interactomes of the proteins of several influenza virus strains (35). Interestingly, among the 132 host factors found in association with PB2 in this study, 29 bind to UPS targets of PB2 detected in our study. These observations highlight the complementary nature of the two interactomics strategies; while mass spectrometry approaches characterize proteins in complexes, systematic binary PPI screenings point to direct targets within the host proteome. Both of these strategies contributed to the identification of host factors functionally relevant to IAV infection (35).

Indeed, of the 41 top-confidence PB2 interactors identified in our screening, a total of 36 were shown by siRNA-based experiments to affect IAV replication, with the depletion of 13, 25, and 31 UPS factors decreasing the production of the H3N2, H1N1_pdm09_, and H1N1_WSN_ viruses, respectively. This high functional output emphasizes the strong ability of this interactomic strategy to detect biologically relevant partners of PB2. The role of these UPS factors seems to be highly virus specific, since only six factors are involved in the replication cycle of all three low- to mild-virulence IAV strains studied. These factors could represent a core set of UPS factors generally involved in the regulation of IAV. They consist of members of E3 ligase complexes based on cullin4 (DCAF12L, DDB1) or cullin1 (FBXL19), a component of the H2A histone ubiquitin ligase complex (PHC2), and DUBs (OTUD6A, PAN2). DDB1 has pleiotropic effects by acting as an adapter of multiple cullin4-based E3 ligase complexes with different SRFs, and the DCAF12L1 SRF has only one documented targeted substrate protein, SH2 domain-containing protein 2A. The SRF of cullin1-based complex FBXL19 has been shown to target the receptor of interleukin-33 (IL-33), ST2L, for degradation, thereby limiting the inflammatory effects of IL-33 (36). Its association with the PB2 protein could alter inflammation in response to IAV infection. Alternatively, we suspect that the binding of PB2 to the SRF of CRL ligases could rewire the E3 ligase complex toward novel cellular or viral factors for ubiquitination. The PCH2 protein is a component of a complex required to maintain the transcriptionally repressive state of many cellular genes. Its interaction with PB2 might be involved in the colocalization of the viral RNPs to inactive chromatin that has been detected at late times of IAV infection (37). The OTUD6A DUB hydrolyzes all ubiquitin chains but the K48-linked chains but has been barely described from a functional point of view, while PAN2 is an inactive DUB with a huge panel of interacting partners in PPI databases, which could therefore act through binding to other factors.

For the remaining 30 functionally relevant UPS factors, a differential effect was observed from strain to strain, highlighting an unexpectedly diversified functional interplay between IAV and the UPS. Strain-specific disparities may provide clues to the molecular mechanisms underlying differences in pathogenicity, while conserved effects could serve as targets for broad-spectrum therapeutics.

The strain specificity of the virus-host interplay can be determined by comparing the interaction profiles of viral factors against the same set of host proteins (32, 33). We showed by agglomerative hierarchical clustering that the PB2-UPS interaction patterns are segregating according to the lineage of origin of the PB2 segment, as well as to the duration of its circulation in the human population. It has to be noted that laboratory-adapted strain H1N1_WSN_ has been grown in cell lines since it was isolated from a human patient in 1933. Its current virulence in humans is therefore not defined, but it is considered a low-pathogenicity strain. The *ex vivo* passages in cell lines do not reflect virus circulation in the human population, so that this strain has been considered in our study only according to its circulation time in humans. The position of the purely avian PB2 away from the other human-infecting avian strains in the interaction dendrogram, which suggests that PB2-UPS interaction profiles might be relevant for detection of the ability of avian strains to infect humans before they acquire human-to-human transmission potential. Therefore, the PB2-UPS interplay may help predict the potential of IAV strains emerging from avian reservoirs to infect humans. The UPS of *Gallus gallus* appears, judging from the UUCD database, to be less complex than the human UPS (5). It is thus plausible that an optimization of the virus interplay with the human UPS takes part in the adaptation of avian strains to humans. Analysis of the PB2-UPS interaction for a larger number of human and avian strains collected at different time points is needed to support this possibility.

The UPS-dedicated comparative interactomic strategy thus constitutes a valuable resource available to be applied to other pathogens to decipher UPS-pathogen protein interplay that could also provide valuable insights into significant pathogen traits.

## MATERIALS AND METHODS

### Plasmids.

Gateway-compatible destination GPCA (pDEST) vectors pSPICA-N1 and pSPICA-N2 were both derived from the pCiNeo mammalian expression vector and expressed, respectively, the Glc1 and -2 complementary fragments of the *G. princeps* luciferase linked to the N-terminal ends of the proteins tested after recombinatorial cloning (Gateway Cloning System; Invitrogen). The open reading frames (ORFs) encoding PB2 from influenza viruses A/WSN/33 (H1N1_WSN_), A/Bretagne/7608/2009 (H1N1_pdm09_), A/Centre/1003/2012 (H3N2), A/Anhui/1/2013 (H7N9), A/BrevigMission/1/1918 (H1N1_1918_), and A/Mallard/Marquenterre/Z2371/83 (H1N1_MZ_) were cloned into Gateway donor vector pDONR207, and the resulting entry clones were transferred into Gateway pDEST vector pSPICA-N2 to produce the Glc2-PB2-expressing plasmids. The ORFs encoding UPS factors were obtained as Gateway entry plasmids from the human ORFeome (the CCSB human ORFeome collection) and transferred into Gateway pDEST pSPICA-N1 to obtain Glc1-UPS-expressing plasmids. The RRS contains human ORFs encoding the following proteins randomly picked from the human ORFeome and *a priori* not interacting with viral protein PB2: LRCC28, NXP2, NFE2L1, GSTT1, GYPA, DPYSL2, UGT3A1, DBH, PLEKHA9, NXPH1, CNTN2, SLC7A13, and CACNG7. The PRS corresponds to human ORFs encoding the NFX1, RUVBL2, NUP50, PTGES3, and KPNA2 proteins picked up from the human ORFeome, which have been shown in the literature to bind PB2.

### Cell lines.

HEK-293T and A549 cells were grown in Dulbecco’s modified Eagle’s medium supplemented with 10% fetal calf serum (FCS). MDCK-SIAT cells were grown in modified Eagle’s medium supplemented with 5% FCS.

### HT-GPCA.

HEK-293T cells were seeded into white 96-well plates at 3 × 10^4^/well. After 24 h, cells were transfected with linear PEI (polyethylenimine) with 300 ng of a Glc2-PB2-expressing plasmid and 100 ng of a Glc1-UPS-expressing plasmid. At 24 h posttransfection, cells were washed with 100 µl of phosphate-buffered saline and lysed with 40 µl of *Renilla* lysis buffer (Promega E2820) for 1 h. *G. princeps* luciferase enzymatic activity was measured with a Berthold Centro LB960 luminometer by injecting 50 µl of luciferase substrate reagent (Promega E2820) per well and measuring luminescence for 10 s. Results were expressed in relative luminescence units.

### NLR retesting.

For the NLR method, the Glc2-PB2/Glc1-UPS pairs were tested in HT-GPCA along with controls consisting of 300 ng of Glc2-PB2 plus 100 ng of Glc1 and 300 ng of Glc2 plus 100 ng of Glc1-UPS. The NLR was calculated as the fold change normalized over the sum of the controls. For a given protein pair A and B, NLR = (Glc1-A + Glc2-B)/[(Glc1-A + Glc2) +(Glc1 + Glc2-B)]. Retesting experiments were conducted three times for each UPS factor.

### Viruses.

The A/WSN/33(H1N1) virus was produced by reverse genetics as described in reference 38. The recombinant A/WSN/33 virus expressing mCitrine was produced by reverse genetics with a pPolI-PB2 plasmid with a sequence encoding the 2A peptide from porcine teschovirus, followed by the mCitrine coding sequence, as described in reference 39 for the nanoluciferase ORF. Influenza viruses A/Centre/1003/2012(H3N2) and A/Bretagne/7608/2009(H1N1_pdm09_) were provided by the National Influenza Center at the Institut Pasteur (Paris, France) and passaged five times on A549 cells at a multiplicity of infection (MOI) of 0.01 PFU/cell. After the fifth passage, there was a >2-log increase in the titers of adapted viruses produced on A549 cells. The hemagglutinins (HAs) of adapted virus strains H1N1_pdm09_ and H3N2 were sequenced. One mutation in the H3N2 HA (G460T) and two mutations in the H1N1_pdm09_ HA (A517G and G834A) were identified. They were introduced into the HA-encoding plasmid in the A/Centre/1003/2012(H3N2) and H1N1A/Bretagne/7608/2009(H1N1_pdm09_) reverse genetics system to produce A549-adapted virus strains H3N2 and H1N1_pdm09_.

### siRNA assays.

siRNAs were purchased from Dharmacon (ON-TARGETplus SMARTpools and Nontargeting Control pool). A549 cells were transfected with 25 nM siRNA with the DharmaFECT1 transfection reagent (Dharmacon). At 48 h posttransfection, cells were infected with the H1N1_WSN_ (MOI, of 0.0001 PFU/cell) or adapted H3N2 and H1N1_pdm09_ (MOI, 0.001 PFU/cell) virus for 24 h. Plaque assays with MDCK-SIAT cells were performed as described in reference 40.

Knockdown efficiency of siRNA was monitored by using expression plasmids for UPS factors fused to *G. princeps* luciferase as described in reference 41, except for DCAF12L1, for which knockdown efficiency was monitored by using quantitative reverse transcription (qRT)-PCR. For this factor, cell lysates were harvested and subjected to total RNA extraction with the RNeasy minikit (Qiagen). qRT-PCR was conducted with forward primer 5′ AATGCGCTCTACACCCACTG 3′ and reverse primer 5′ TTGACCAAAGGACCACTCACT 3′ by using the protocol of the LightCycler RNA amplification kit SYBR green I (Roche). The cellular glyceraldehyde-3-phosphate dehydrogenase mRNA in the infected cells was also quantified as an internal control by using the above-described qRT-PCR. The DCAF12L1 siRNA decreased the level of DCAF12L1 mRNA by 50% (not shown). Cell viability was determined by trypan blue counting at 48 h posttransfection with the siRNAs at 25 nM.

### Overexpression-based experiments.

HEK-293T cells were seeded into the wells of white 96-well plates at 3 × 10^4^/well. After 24 h, cells were transfected with linear PEI and 150 ng of plasmid pCINeo expressing a UPS factor fused to mCherry. At 24 h posttransfection, cells were infected with the WSN-PB2-2A-mCitrine virus at an MOI of 1 PFU/cell for 18 h. Cells were then fixed with a 4% paraformaldehyde solution and analyzed by flow cytometry (Attune NxT; Thermo Fisher Scientific).

### Statistics. (i) Whisker plot representation.

The distributions of the relative luminescence values are represented by whisker plot boxes. Whisker length corresponds to 1.5 times the interquartile range (IQR) that is equal to the difference between the upper and lower quartiles (IQR = Q3 − Q1). The upper whisker, defined by the third quartile (Q3) plus 1.5 times the IQR, was defined as the PT as follows: PT = Q3 + 1.5(IQR). The R software (40) was used to make calculations with the boxplot function. The scripts used are available upon request.

### (ii) Determination of the confidence interval.

To estimate the significance of an NLR for a given protein-protein pair by comparison to the RRS sampling signal, a confidence interval was calculated for the RRS data set, considering the estimated standard error (SE) and a confidence level of 99.73% (i.e., a risk α of 0.27%) by using the expression (μ − *t*.SE) + (μ + *t*.SE), where *t* is the critical value for a two-sided Student test and for *n* − 1 degrees of freedom. In a normally distributed population, a 99.73% confidence interval corresponds to the mean ± 3 standard deviations. We considered the NLR of a new sample to be statistically significantly different from the RRS if its value was larger than the upper bound of the confidence interval determined for the RRS data set. The R software (40) was used to make calculations with the t.test function. The scripts used are available upon request.

### (iii) Hierarchical clustering.

NLRs for each PB2 were normalized by using *z* scores calculated as follows: *z* score = (NLR − mean NLR for PB2)/standard deviation. The distance matrix was calculated on the basis of *z* scores with the R software (40) by using euclidian distance parameter of the dist function. Clustering was then performed by the classical ward.D method with the R software (42) by using the hclust function. The scripts used are available upon request.
